# Influence of CYP2D6 polymorphisms on tamoxifen side effects in patients with breast cancer

**DOI:** 10.1007/s12094-025-03908-y

**Published:** 2025-04-11

**Authors:** Isabel Blancas, Marina Linares-Rodríguez, Carlos José Rodríguez-González, Fernando Rodríguez-Serrano

**Affiliations:** 1 Department of Medicine, University of Granada, Granada, Spain; 2 Section of Medical Oncology, Hospital Universitario Clínico San Cecilio, Granada, Spain; 3 Instituto de Investigación Biosanitaria ibs.GRANADA, Granada, Spain; 4 Biopathology and Regenerative Medicine Institute (IBIMER), University of Granada, Granada, Spain; 5 Department of Human Anatomy and Embryology, University of Granada, Granada, Spain

**Keywords:** Breast cancer, Tamoxifen, CYP2D6, Side effects, Uterine changes

## Abstract

**Purpose:**

CYP2D6 is a key enzyme involved in converting tamoxifen into its active metabolites. However, polymorphisms in CYP2D6 lead to variable enzymatic capacities. We aimed to examine the impact of CYP2D6 polymorphisms on tamoxifen-derived side effects in breast cancer patients.

**Methods:**

Eighty-six patients with hormone receptor–positive breast cancer who received tamoxifen were classified as poor (PM), intermediate (IM), normal (NM), or ultrarapid (UM) metabolizers according to Clinical Pharmacogenetics Implementation Consortium (CPIC) guidelines. All patients received 20 mg/day tamoxifen for 5 years, except PM, who were dose-escalated (20 mg/day for 4 months, 40 mg/day for 4 months, 60 mg/day for 4 months, then back to 20 mg/day). Adverse events—osteoarticular pain, hot flashes, asthenia, and uterine changes—were analyzed by Kaplan–Meier and Cox regression. A propensity score–matched (PSM) subgroup was also examined.

**Results:**

Rapid metabolizers (RM: NM + UM) consistently showed fewer uterine changes compared to slow metabolizers (SM: PM + IM) in both the entire cohort (HR 0.20, p = 0.001) and the PSM subgroup (HR 0.07, p = 0.011). Excluding PM and UM, comparison of IM vs. NM showed similar differences (complete group: HR 0.20, p = 0.002; PSM subgroup: HR 0.23, p = 0.068). Other side effects (joint pain, hot flashes, asthenia) were not significantly associated with CYP2D6 phenotype.

**Conclusion:**

Uterine alterations in breast cancer patients treated with tamoxifen appear linked to decreased CYP2D6 activity, although we observed no association between CYP2D6 and other toxicities. These findings suggest closer monitoring for uterine toxicity in individuals with impaired CYP2D6 metabolism.

## Introduction

Breast cancer remains a major global health issue due to its high incidence and mortality rates. It is the most frequently diagnosed cancer among women worldwide, with 2.3 million new cases in 2022, comprising 11.6% of all cancer diagnoses, and ranks as the fourth leading cause of cancer-related deaths [1]. Approximately 80% of breast cancers express estrogen receptors (ER) [2], for which tamoxifen is frequently employed. Tamoxifen is a selective estrogen receptor modulator that can exhibit agonist or antagonist activities depending on the target tissue. In breast tissue, it blocks estrogen-driven cell proliferation [3]. Beyond ER-positive (ER +) tumors, tamoxifen may benefit progesterone receptor–positive (PR +) tumors [4], including some that are PR + but seemingly lack ER [3].

Tamoxifen is metabolized within the cytochrome P450 system, with CYP2D6 playing a pivotal role by converting tamoxifen to potent metabolites such as 4-hydroxytamoxifen and endoxifen. Endoxifen has been described as up to 100 times more potent in estrogen antagonism than tamoxifen itself [5–8]. Patients harboring CYP2D6 polymorphisms resulting in low or absent CYP2D6 activity, or those receiving CYP2D6 inhibitors concurrently, exhibit substantially lower endoxifen concentrations [9, 10]. Variation in CYP2D6 results in different metabolic phenotypes, including poor (PM), intermediate (IM), normal (NM), and ultrarapid (UM) metabolizers [11, 12]. Our previous work indicated that PM patients have up to fourfold lower endoxifen and 4-hydroxytamoxifen concentrations compared with NMs [13]. Despite tamoxifen efficacy in reducing breast cancer recurrence and mortality, it can cause side effects such as hot flashes, uterine changes, osteoarticular pain, asthenia, and others [14–20].

The present study aimed to evaluate long-term tamoxifen toxicity in patients harboring distinct CYP2D6 metabolizer phenotypes. We analyzed both the entire trial cohort and a propensity score–matched (PSM) subset to control for confounders. Because PM patients received a dose-escalation strategy, we also carried out separate comparisons between IM and NM subgroups to evade the potential confounding effect of dose escalation.

## Patients and methods

### Patients

A total of 86 patients diagnosed with breast cancer at San Cecilio University Hospital (Granada, Spain) were enrolled. All had hormone receptor–positive (ER + and/or PR +) breast cancer and were scheduled to receive tamoxifen (20 mg/day) for at least 5 years, except for PM patients, who underwent a dose-escalation scheme: 20 mg/day for 4 months, 40 mg/day for 4 months, 60 mg/day for 4 months, then reverting to 20 mg/day for the remainder of the treatment course. The mean follow-up period was 139.5 months (95% CI 133.0–144.9). Clinical data (e.g., age, histological grade, nodal status, tumor size, HER2 status, Ki-67, prior chemotherapy, radiotherapy) and toxicity outcomes were collected.

The study was approved by the Clinical Research Ethics Committee of Hospital Universitario San Cecilio and the Spanish Agency of Medicines and Medical Devices. It was registered in the European Union Clinical Trials Register (EudraCT Number: 2007–002942-40). All participants provided written informed consent.

### Genotyping of CYP2D6 polymorphisms

Initially, 10 mL of peripheral blood were collected from each participant, and a 200 µL aliquot of this sample was used for DNA isolation. DNA extraction was performed using the QIAcube automated system kit (Qiagen), following manufacturer guidelines. DNA quantity was measured by UV spectrophotometry (Jenway-Genova) at 260 nm, considering that one absorbance unit corresponds approximately to 50 ng/µL of double-stranded DNA. DNA purity was assessed using the A260/A280 absorbance ratio, considering optimal values between 1.8 and 2.0. Values below 1.8 suggest potential protein contamination. CYP2D6 genotypes were determined using the AmpliChip CYP450 test (Roche Diagnostics, Indianapolis, IN, USA), a widely validated method for detecting clinically relevant CYP2D6 variants [13, 21, 22]. This test was chosen because it detects key CYP2D6 variants influencing tamoxifen metabolism. Phenotypes were classified according to the CYP2D6 activity score (AS) of the Clinical Pharmacogenetics Implementation Consortium (CPIC) guidelines: PM (AS = 0), IM (0 < AS < 1.25), NM (1.25 ≤ AS ≤ 2.25), UM (AS > 2.25) [23, 24]. For certain analyses, PM + IM were combined as SM (slow metabolizers), whereas NM + UM were combined as RM (rapid metabolizers). Additionally, to avoid potential confounding by dose escalation in PM, we performed a separate analysis restricted to IM vs. NM. Evaluations were conducted both in the complete cohort and in a PSM-matched subgroup.

### Statistical analysis

Baseline characteristics were reported as frequencies and percentages. Differences in categorical variables were tested using chi-square or Fisher exact tests, as appropriate. To minimize confounding, we performed 1:1 propensity score matching (PSM) using logistic regression and the greedy matching method [25] in R (version 4.2.2). Variables used for matching included tumor grade, tumor size, patient age, nodal status, and receipt of chemotherapy/radiotherapy.

Kaplan–Meier analysis was used to estimate time-to-event for each adverse effect—osteoarticular pain, hot flashes, asthenia, and uterine changes. Uterine changes included a variety of conditions, ranging from benign to serious pathologies. These included endometrial hyperplasia, polyps, adenocarcinoma, thickened myometrium or endometrium, and multiple fibroids. The Cox proportional hazards model yielded hazard ratios (HR) with 95% confidence intervals. Significance was set at p < 0.05. Data were analyzed with IBM SPSS Statistics v28 (SPSS, Inc., Chicago, IL).

## Results

### Patient characteristics

Of the 86 total participants, 51 were classified as RM (NM or UM) and 35 as SM (PM or IM). After PSM, 21 RM and 21 SM patients remained. No significant baseline differences were noted between RM and SM in either the full or the matched subset. Most participants were under 50 years old. Grade II tumors predominated, and node-negative disease was most common. Nearly all tumors (93–95%) were ER + or PR + . Most patients received adjuvant chemotherapy (primarily anthracyclines ± taxanes) and/or radiotherapy (Table 1).

**Table 1:** Clinical and pathological variables of all patients and the PSM subgroup

		All Patients			PSM Paired		
Characteristic	Group	RM (*n* = 51)	SM (*n* = 35)	p.value	RM (*n* = 21)	SM (*n* = 21)	p.value
Age	< 50	39 (76.5)	30 (85.7)	0.410	18 (85.7)	19 (90.5)	1.000
	≥ 50	12 (23.5)	5 (14.3)		3 (14.3)	2 ( 9.5)	
Tumor Grade	I	5 (12.5)	3 (12.0)	1.000	3 (14.3)	3 (14.3)	1.000
	II	29 (72.5)	18 (72.0)		17 (81.0)	17 (81.0)	
	III	6 (15.0)	4 (16.0)		1 ( 4.8)	1 ( 4.8)	
Nodal Status	N0	21 (48.8)	18 (58.1)	0.390	13 (61.9)	13 (61.9)	1.000
	N1-3	16 (37.2)	7 (22.6)		4 (19.0)	4 (19.0)	
	N4/ +	6 (14.0)	6 (19.4)		4 (19.0)	4 (19.0)	
Tumor Size	≤ 2	26 (63.4)	16 (50.0)	0.340	11 (52.4)	12 (57.1)	1.000
	> 2 < 5	15 (36.6)	16 (50.0)		10 (47.6)	9 (42.9)	
ER	Negative	3 ( 6.1)	2 ( 6.1)	1.000	1 ( 4.8)	1 ( 4.8)	1.000
	Positive	46 (93.9)	31 (93.9)		20 (95.2)	20 (95.2)	
PR	Negative	5 (10.2)	2 ( 6.1)	0.696	1 ( 4.8)	1 ( 4.8)	1.000
	Positive	44 (89.8)	31 (93.9)		20 (95.2)	20 (95.2)	
HER2	Negative	37 (80.4)	28 (84.8)	0.768	16 (84.2)	16 (80.0)	1.000
	Positive	9 (19.6)	5 (15.2)		3 (15.8)	4 (20.0)	
Radiotherapy	No	14 (27.5)	10 (28.6)	1.000	7 (33.3)	6 (28.6)	1.000
	Yes	37 (72.5)	25 (71.4)		14 (66.7)	15 (71.4)	
Chemotherapy	No	12 (23.5)	6 (17.1)	0.593	4 (19.0)	3 (14.3)	1.000
	Yes	39 (76.5)	29 (82.9)		17 (81.0)	18 (85.7)	
Type	None	12 (23.5)	6 (17.1)	0.725	4 (19.0)	3 (14.3)	1.000
	Ant	9 (17.6)	9 (25.7)		5 (23.8)	6 (28.6)	
	Ant. + Taxanes	29 (56.9)	20 (57.1)		12 (57.1)	12 (57.1)	
	Other	1 ( 2.0)	0 ( 0.0)				

### Genotype and phenotype of CYP2D6

Analysis of the CYP2D6 gene revealed *1, *2, and *4 as the most frequent alleles (74.4% combined). The most common genotypes were *1/*2, *1/*1, and *1/*4. In the full cohort, 5.8% were PM, 34.9% IM, 58.1% NM, and 1.2% UM. In the PSM subset (42 patients), 11.9% were PM, 38.1% IM, and 50% NM; no UM phenotypes were observed. Table 2 and Table 3 summarize the genotype frequencies and phenotype distributions.

**Table 2:** CYP2D6 allelic and phenotypic frequency

CYP2D6 allele	*n* (%)	Genotype	*n* (%)	Genotype	*n* (%)
*1	56 (32.6)	*1/*2	12 (14)	*9/*41	2 (2.3)
*2	37 (21.5)	*1/*1	11 (12.8)	*10/*35	1 (1.2)
*4	35 (20.3)	*1/*4	8 (9.3)	*17/*41	1 (1.2)
*35	12 (7.0)	*1/*35	6 (7)	*1XN/*2	1 (1.2)
*41	11 (6.4)	*2/*2	6 (7)	*2/*35	1 (1.2)
*9	9 (5.2)	*2/*4	6 (7)	*2/*41	1 (1.2)
*10	7 (4.1)	*4/*4	5 (5.8)	*2/*5	1 (1.2)
*17	2 (1.2)	*4/*35	4 (4.7)	*2/*9	1 (1.2)
*1XN	1 (0.6)	*1/*41	3 (3.5)	*2XN/*4	1 (1.2)
*5	1 (0.6)	*1/*9	3 (3.5)	*4/*17	1 (1.2)
*2XN	1 (0.6)	*1/*10	2 (2.3)	*4/*9	1 (1.2)
		*2/*10	2 (2.3)	*41/*41	1 (1.2)
		*4/*10	2 (2.3)	*9/*9	1 (1.2)
		*4/*41	2 (2.3)

**Table 3:** AS values and CYP2D6 phenotype frequency

		All Patients	PSM Paired
CYP2D6 phenotype	AS* value per genotype	*n* (%)	
*Poor (PM)*	0	5 (5.8)	5 (11.9)
*Intermediate (IM)*	0 < x < 1.25	30 (34.9)	16 (38.1)
*Normal (NM)*	1.25 ≤ x ≤ 2.25	50 (58.1)	21 (50.0)
*Ultrarapid (UM)*	> 2.25	1 (1.2)	0 (0)

^*^AS: Activity score according to the Clinical Pharmacogenetics Implementation Consortium (CPIC)

### Influence of CYP2D6 polymorphism on adverse effects

#### Complete cohort

**Fig. 1:**
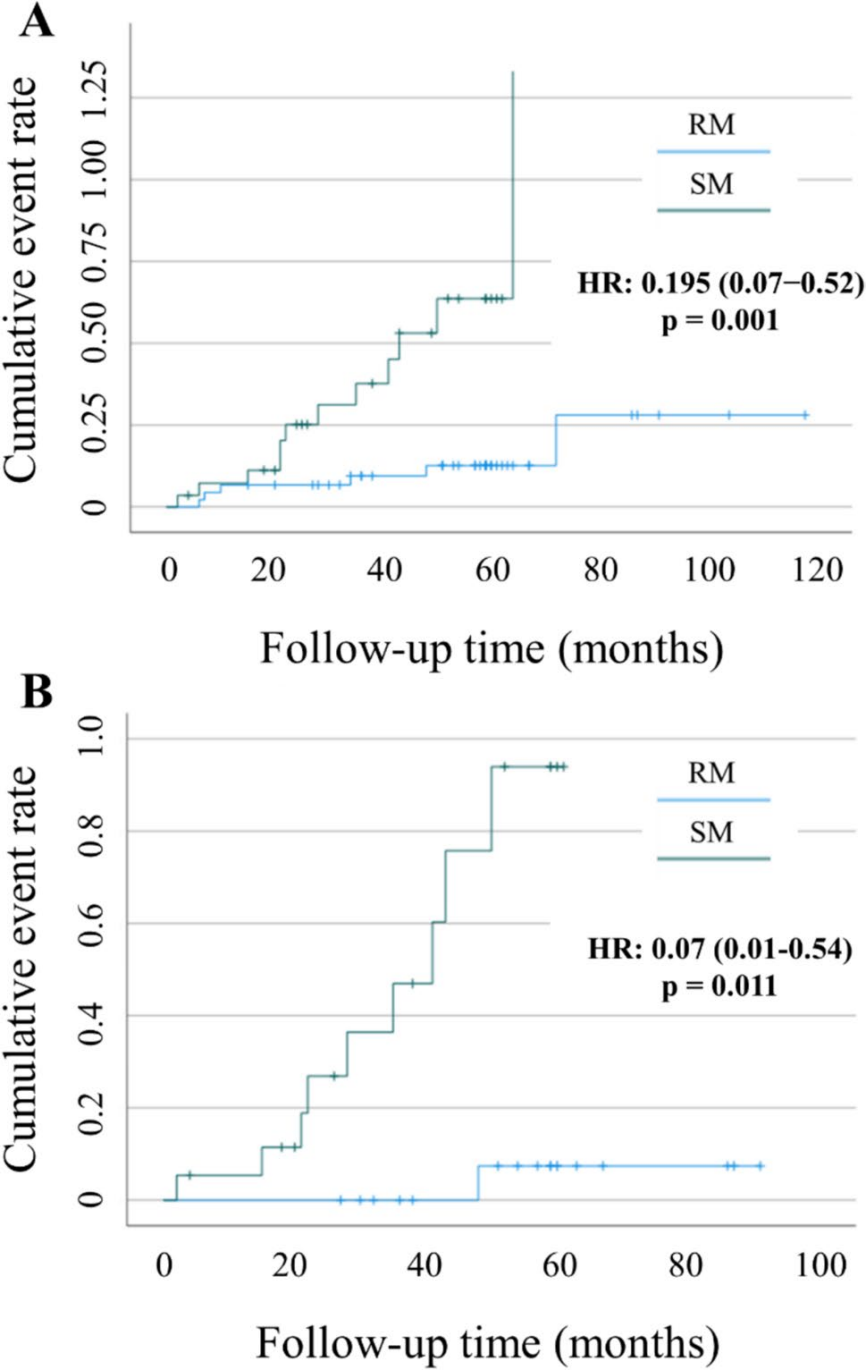
Cumulative incidence of uterine changes stratified by (**A)** rapid metabolizers (RM) vs. slow metabolizers (SM) in the complete cohort, and (**B)** RM vs. SM in the PSM-matched subgroup

We examined osteoarticular pain, hot flashes, asthenia, and uterine changes in RM vs. SM (Table 4, Fig. 1). Osteoarticular pain occurred in 60% of RM vs. 50% of SM (p = 0.566), hot flashes in 47.8% of RM vs. 36.7% of SM (p = 0.526), and asthenia in 40% of RM vs. 51.7% of SM (p = 0.164). However, uterine changes were significantly more frequent in SM (44.8%) vs. RM (15.2%) (p < 0.001), with an HR of 0.20 (p = 0.001).

**Table 4:** Adverse effects associated with RM and SM in the complete cohort

	*N*	Events (%)	Time to Events (months; CI 95%)	*P*	HR (CI 95%)	*P*
*Osteoarticular pain*						
RM	45	27 (60)	56.5 (38.3 − 74.7)	0.566	1.20 (0.63 − 2.29)	0.571
SM	30	15 (50)	37.9 (29.0 − 46.8)		Reference	
*Hot flashes*						
RM	46	22 (47.8)	83.4 (59.9 − 106.9)	0.526	1.26 (0.61 − 2.62)	0.533
SM	30	11 (36.7)	43.4 (33.3 − 53.5)		Reference	
*Asthenia*						
RM	45	18 (40)	74.2 (58.1 − 90.2)	0.164	0.62 (0.31 − 1.24)	0.176
SM	29	15 (51.7)	32.7 (22.2 − 43.2)		Reference	
*Uterine changes*						
RM	46	7 (15.2)	113.7 (94.7 − 132.8)	**0.000**	0.20 (0.07 − 0.52)	**0.001**
SM	29	13 (44.8)	55.3 (38.9 − 71.8)		Reference	

Bold values indicate statistical significance

All values are n (%)

To account for potential confounding from PM dose escalation, a separate analysis was conducted comparing only NM vs. IM (Table 5). Similar findings emerged for uterine changes: 15.6% of NM vs. 48% of IM (p < 0.001; HR = 0.20, p = 0.002), while other side effects showed no significant differences (p > 0.05).

**Table 5:** Adverse effects associated with NM and IM in the complete cohort

	*N*	Events (%)	Time to Events (months; CI 95%)	*P*	HR (CI 95%)	*P*
*Osteoarticular pain*						
NM	44	27 (61.4)	54.9 (36.7—73.2)	0.465	1.28 (0.65—2.51)	0.471
IM	26	13 (50)	38.3 (28.7—47.8)		Reference	
*Hot flashes*						
NM	45	22 (48.9)	81.2 (57.3—105.1)	0.410	1.38 (0.63—3.02)	0.418
IM	26	9 (34.6)	44.6 (33.8—55.3)		Reference	
*Asthenia*						
NM	44	18 (40.9)	73.0 (56.7—89.4)	0.331	0.70 (0.33—1.46)	0.342
IM	25	12 (48)	35.0 (23.7—46.3)		Reference	
*Uterine changes*						
NM	45	7 (15.6)	113.4 (94.3—132.6)	** < 0.001**	0.20 (0.07—0.54)	**0.002**
IM	25	12 (48)	55.8 (38.9—72.7)		Reference	

Bold values indicate statistical significance

NM: Normal metabolizers; IM: Intermediate metabolizers

#### PSM-matched cohort 

In the matched cohort, no significant RM vs. SM differences were observed for osteoarticular pain, hot flashes, or asthenia (p > 0.05). Yet uterine changes again showed a pronounced discrepancy: 5.3% in RM vs. 47.4% in SM (p = 0.001), HR 0.07 (p = 0.011) (Table 6, Fig. 1). Similarly, restricting the analysis to NM vs. IM in the PSM subset (Table 7) indicated a higher incidence of uterine events among IM (43.7%) vs. NM (13.3%), although the difference narrowly missed significance in the Cox model (p = 0.068).

**Table 6:** Adverse effects associated with RM and SM in the PSM cohort

	*N*	Events (%)	Time to Events (months; CI 95%)	*P*	HR (CI 95%)	*P*
*Osteoarticular Pain*						
RM	18	9 (50)	79.0 (51.0–107.1)	0.439	0.69 (0.27–1.77)	0.443
SM	20	10 (50)	36.8 (26.8–46.8)		Reference	
*Hot flashes*						
RM	19	10 (52.6)	86.2 (50.4–122.0)	0.938	1.04 (0.41–2.62)	0.939
SM	20	9 (45)	39.2 (26.9–51.4)		Reference	
*Asthenia*						
RM	17	8 (47.1)	72.7 (45.8–99.6)	0.245	0.58 (0.22–1.49)	0.256
SM	20	11 (55.0)	30.9 (18.7–43.1)		Reference	
*Uterine changes*						
RM	19	1 (5.3)	87.9 (82.1–93.7)	**0.001**	0.07 (0.01–0.54)	**0.011**
SM	19	9 (47.4)	42.4 (33.3–51.4)		Reference	

Bold values indicate statistical significance

*RM:* Rapid metabolizers, *SM:* Slow metabolizers

**Table 7:** Adverse effects associated with NM and IM in the in the PSM cohort

		*N*	Events (%)	Time to Events (months; CI 95%)	*P*	HR (CI 95%)	*P*
*Osteoarticular pain*							
NM	15		7 (46.7)	51.8 (32.9—70.6)	0.985	0.99 (0.36—2.74)	0.985
IM	17		8 (47.1)	39.7 (29.0—50.4)		Reference	
*Hot flashes*							
NM	15		8 (53.3)	31.3 (17.7—44.8)	0.453	1.47 (0.53—4.06)	0.460
IM	17		7 (41.2)	41.7 (28.6—54.8)		Reference	
*Asthenia*							
NM	14		8 (57.1)	29.6 (16.0—43.3)	0.602	1.30 (0.47—3.61)	0.609
IM	16		7 (43.7)	37.9 (24.4—51.3)		Reference	
*Uterine changes*							
NM	15		2 (13.3)	78.3 (66.7—89.9)	**0.047**	0.23 (0.05—1.12)	0.068
IM	16		7 (43.7)	46.0 (37.0—55.1)		Reference	

Bold values indicate statistical significance

NM: Normal metabolizers, IM: Intermediate metabolizers

### Relationship between uterine changes and clinical characteristics

Multivariate analysis found that reduced CYP2D6 enzymatic capacity was the principal factor associated with uterine changes in both the entire group (HR 0.10, p = 0.004) and the PSM cohort (HR 0.04, p = 0.004). Other variables such as tumor size, grade, nodal status, chemotherapy, or radiotherapy did not show a significant correlation with uterine toxicity (Table 8).

**Table 8:** Multivariate analysis in the entire cohort and the PSM group regarding uterine changes

	All Patients		PSM Paired	
Multivariate analysis	HR (CI 95%)	*P*	HR (CI 95%)	*P*
*Uterine Changes*				
CYP2D6 (RM vs **SM**)	0.10 (0.02–0.47)	**0.004**	0.04 (0.00–0.36)	**0.004**
Tumour size (≤ 2 vs > **2 < 5**)	0.47 (0.11–2.02)	0.311	0.30 (0.06–1.57)	0.154
Grade (I vs **III**)	3.68 (0.2–67.39)	0.379	0.05 (0.00–2.25)	0.122
Grade (II vs **III**)	2.05 (0.19–21.55)	0.552	0.10 (0.00–2.52)	0.161
Radiotherapy (No vs **Yes**)	0.83 (0.19–3.54)	0.797	0.24 (0.03–2.16)	0.202
Chemotherapy (No vs **Yes**)	0.50 (0.04–5.82)	0.583	0.87 (0.07–11.2)	0.913
Nodal (N0 vs **N ≠ 0**)	0.77 (0.19–3.09)	0.714	0.66 (0.12–3.61)	0.628

The reference group for comparisons is shown in bold

## Discussion

Tamoxifen substantially decreases recurrence risk and mortality in ER + breast cancer [3]. It requires hepatic metabolism via CYP2D6 to form key active metabolites, including endoxifen [10, 23]. Genetic differences in CYP2D6 can lead to suboptimal tamoxifen efficacy in PM patients, whereas UMs may face excessive toxicity [26].

In this study, we implemented an early tamoxifen dose-escalation regimen in PM patients (20 → 40 → 60 mg/day) to boost endoxifen levels, consistent with prior reports demonstrating that elevated doses can improve drug exposure [22, 27–29]. Nonetheless, we evaluated whether polymorphisms in CYP2D6 might also influence adverse-event profiles [14, 16, 18, 20, 30]. Our data strongly implicate lower CYP2D6 activity as a driver of heightened uterine toxicity, whereas no significant differences emerged for hot flashes, asthenia, or osteoarticular pain. Specifically, SM phenotypes (PM + IM) showed a higher incidence of uterine changes compared to RM (NM + UM). Excluding PM (who received higher doses) and focusing on IM vs. NM yielded similar patterns, suggesting that poor metabolic function—not necessarily dose escalation—underlies the observed increase in uterine adverse events.

Studies have long noted that tamoxifen may exert partial estrogen agonism in the uterus, raising risks of endometrial proliferation, hyperplasia, and malignancies [31–35]. Some investigators report that PM patients harbor higher concentrations of certain tamoxifen metabolites that may amplify estrogenic effects in endometrial tissue [36, 37].

In our study, differences in hot flashes or joint pains did not reach significance. Others have reported mixed findings regarding a link between CYP2D6 genotype and hot flashes [38–40]. Although arthralgia is recognized as a common tamoxifen side effect [14–17], we found no clear correlation with genotype.

Because of the limited number of PM and UM patients, further research is warranted in larger cohorts, ideally with direct endoxifen quantification. Our data, however, suggest that dose escalation in PM does not itself systematically heighten toxicity; conversely, many IM patients receiving standard doses exhibited significant uterine changes. These observations align with other studies reporting that higher tamoxifen doses can increase endoxifen levels without invariably worsening toxicity [41, 42].

## Conclusion

In summary, the present findings underscore an association between reduced CYP2D6 activity (PM or IM) and an elevated risk of uterine alterations in tamoxifen-treated breast cancer patients. By contrast, no significant relationship emerged between CYP2D6 polymorphisms and other adverse events, including joint pain, hot flashes, or asthenia. While PM patients underwent dose escalation, the increased risk of gynecological toxicity was also observed in IM patients at standard doses, suggesting that compromised CYP2D6 metabolism rather than higher tamoxifen doses was primarily responsible. Future large-scale research is necessary to confirm these results and to refine tamoxifen dosing strategies based on CYP2D6 phenotype and / or the protocolized gynecological follow-up.

## Data Availability

The datasets generated and analyzed during the current study are available from the corresponding authors on reasonable request.

## Abbreviations

*AS*: Activity score
*CPIC*: Clinical pharmacogenetics implementation consortium
*ER*: Estrogen receptors
*HR*: Hazard ratio
*IM*: Intermediate metabolizers
*NM*: Normal metabolizers
*PM*: Poor metabolizers
*PR*: Progesterone receptors
*PSM*: Propensity score matching
*RM*: Rapid metabolizers
*SM*: Slow metabolizers
*UM*: Ultra-rapid metabolizers
